# Effects of Printing Temperature and Filling Percentage on the Mechanical Behavior of Fused Deposition Molding Technology Components for 3D Printing

**DOI:** 10.3390/polym13172910

**Published:** 2021-08-29

**Authors:** Ming-Hsien Hsueh, Chao-Jung Lai, Kuan-Yin Liu, Cheng-Feng Chung, Shi-Hao Wang, Chieh-Yu Pan, Wen-Chen Huang, Chia-Hsin Hsieh, Yu-Shan Zeng

**Affiliations:** 1 Department of Industrial Engineering and Management, National Kaohsiung University of Science and Technology, Kaohsiung 807618, Taiwan; mhhsueh@nkust.edu.tw (M.-H.H.); charlie820906@gmail.com (C.-H.H.); tzen010@gmail.com (Y.-S.Z.); 2 Department of Fashion Design and Management, Tainan University of Technology, Tainan 71002, Taiwan; 3 Department and Graduate Institute of Aquaculture, National Kaohsiung University of Science and Technology, Kaohsiung 811213, Taiwan; 4 Department of Information Management, National Kaohsiung University of Science and Technology, Kaohsiung 824005, Taiwan

**Keywords:** polylactic acid, 3D printing, printing temperature, filling percentage, mechanical properties, fused deposition modeling

## Abstract

Additive manufacturing (AM) has the advantages of providing materials with lightweight microporous structures and customized features, and being environmentally safe. It is widely used in medical sciences, the aerospace industry, biological research, engineering applications, and other fields. Among the many additive manufacturing methods, fused deposition modeling (FDM) is relatively low-cost, wastes less raw material and has a lower technical threshold. This paper presents a study on 3D printing based on FDM by changing two printing parameters, namely the printing temperature and filling percentage. The produced polylactic acid (PLA) material was analyzed through tensile and Shore D hardness tests and the differences in mechanical properties before and after the UV curing process were analyzed. The results show that increasing the filling percentage or increasing the printing temperature can effectively improve the tensile Young’s modulus, ultimate tensile strength, elongation, and Shore hardness of the material. The UV curing process could enhance the rigidity and hardness of the material significantly but reduced the strength and toughness of the material. These findings could benefit researchers studying FDM with the goal of achieving sustainable manufactured materials.

## 1. Introduction

Technology is advancing with each passing day. Desktop technologies, including 3D, 4D, 5D and 6D printing technology, also known as additive manufacturing (AM) [1,2,3], has been evolving to meet the requirements of low prices, low consumables, and low operating costs of industry. AM has the advantages of providing lightweight microporous structures with customized features and being environmentally safe. It is widely used in medical sciences, the aerospace industry, biological research, engineering applications, and other fields [4,5,6,7].

In recent years, there has been a lot of research on AM technology, mainly to improve the material properties and control the manufacturing processes [8,9,10]. Additive manufacturing methods include fused deposition modeling (FDM), stereolithography appearance (SLA), selective laser sintering (SLS), etc. Among the many additive manufacturing methods, FDM is relatively low-cost, wastes less material, and has a lower technical threshold, but has higher requirements of control parameters such as nozzle temperature, process speed, filling percentage, material characteristics, etc. [11,12]. These reasons lead to low dimensional accuracy and low strength of objects produced by FDM, which results in a failure to meet traditional manufacturing standards. Thermoplastic materials such as acrylonitrile butadiene styrene (ABS), polylactic acid (PLA), polypropylene (PP) and polyethylene (PE) are more commonly used in extrusion methods, as well as materials that could improve mechanical properties such as polyamide (PA), polycarbonate (PC), polyetheretherketone (PEEK), etc. Compared with other thermoplastic materials, PLA has a lower price, better biocompatibility and biodegradability, higher retrievability, and good mechanical properties [13]. Therefore, it is regarded as an environmentally friendly alternative to petrochemical plastics. During the material-stacking process if it cannot completely stack or completely cure, this results in a decrease in the mechanical properties of the final material. Therefore, post-curing technologies have been developed to perform a secondary curing of the molded material through UV light wavelength irradiation to reduce the unevenness between layers and improve the final mechanical properties. This process is however cumbersome and time-consuming [14,15].

In the past few years, researchers have discussed the impact of printing process parameters and UV curing secondary processing on FDM technology. To improve the performance and quality of the parts, the material structure performance and shortened printing build time must be ensured. Luzanin et al. [16] studied the influence of five construction parameters including layer thickness, deposition angle, filling, extrusion speed, and extrusion temperature on the maximum flexural force of a sample made of PLA and its interactions. DSD statistical analysis revealed that the deposition angle and filler mainly affect the bending characteristics. The filling percentage was set at 10%, 20%, and 30% to reduce the construction time and improve efficiency. Akhoundi et al. [17] investigated that the effect of filling patterns on the tensile and flexural strength and modulus of parts printed via FDM and 3D printers. The result shows that infill percentage and filling pattern are the primary factors affecting the mechanical properties of 3D-printed products. The results indicated that the concentric pattern yields the most desirable tensile and flexural tensile properties, at all filling percentages, apparently due to the alignment of deposited rasters with the loading direction. Hilbert curve patterns also yield a dramatic improvement in the properties at 100% filling. Aloyaydi et al. [18] investigated the influence of infill density on the microstructure and flexural behavior of thermoplastic 3D-printed PLA parts processed by fusion deposition modeling. As the filling percentage increases, the porosity decreases, so that the contact and bonding between the layers increases. This means that the bending characteristics have a high correlation with the filling percentage. Observed in the microstructure, brittle fracture modes (smooth areas) and ductile failure modes (deformation mode) as the filling percentage increase and ductile failure modes (deformation mode) increase. Grasso et al. [19] studied the influence of temperature on the mechanical properties of PLA materials in FDM technology 3D printing. As the temperature increased, the cold crystallization effect of the polymer and the rearrangement of the molecular chains along the test direction increased the stiffness of the material. At higher temperatures, closer to the glass transition temperature of PLA (i.e., 40 °C), this caused stress to redistribute and the failure stress decreased as the temperature increased. Wang et al. [20] found that the FDM in the 3D printing technology is at the high temperature of the nozzle and the heating platform. They observed that the deposited filaments undergo diffusion bonding in the cross-sectional profile and between adjacent filaments. The diffusion between the filaments establishes the surface roughness of the object. The molecular diffusion between filaments affects the cross-sectional area shape and the bonding area of the filaments, which further affects the combination of heat transfer and diffusion. As the nozzle temperature and platform temperature increase, the surface roughness decreases. However, as the temperature rises, the influence of nozzle temperature on the surface roughness of the platform is greater than that of the platform temperature. Using a higher or lower printing speed or a thinner layer thickness deteriorates the surface quality because the nozzle extrusion deteriorates the surface morphology. Behzadnasab et al. [21] discussed the influence of printing temperature, filling pattern, and annealing on the tensile strength and Young’s modulus of PLA by open-source 3D printer technology. The printing conditions have been found to have a significant impact on the final performance of the object. For example, the fracture stress increases from 34 MPa to 56 MPa as the nozzle temperature rises, which is close to the value of 65 MPa seen for injection molded samples. However, the tensile strength increases with the printing temperature, but the annealing process has a negative impact on the mechanical properties of the samples. Tertyshnaya [22] studied the effect of 3D printing technology on the physical properties of composite materials mixed with PLA and low-density polyethylene (LDPE) in FDM materials. They observed that the melting temperature changed by 1–2 °C, and with the glass transition temperature of 70–80 wt%, LDPE increased by 4–5 °C. After irradiating with UV light, LDPE showed brittle failure with the increase in irradiation time. Can et al. [23] investigated that the FDM technology for 3D printing, adding micron and nanometer size TiO_2_ particles and benzotriazole as UV absorbers into PLA. They found that TiO_2_ particles have a strengthening and toughening effect. Due to effective stiffening, strengthening, and toughening actions of TiO_2_ particles, as well as due to their very significant UV screening actions absorbing the photons of the UV irradiation, thus decreasing the degree of the detrimental photodegradation reactions leading to chain scissions in their PLA matrix. The low-temperature aging of PLA material from the molten state to the quenched state of the FDM technology of 3D printing was studied by Zhao et al. [24], to slow down the aging of physical properties and the transformation of entanglement into a secondary ordered structure. UV radiation entangles, and the amorphous chain inhibits the formation of secondary structures during the aging process of physical properties, while the continuous amorphous phase with high entanglement density indicates that the polymer has higher toughness.

To avoid the loss of time and materials, the FDM technology was used in this study to analyze the impact of different filling percentages and nozzle temperatures on the mechanical properties of PLA materials. We also studied whether UV-curing PLA materials could reduce the unevenness between layers and improve the mechanical properties and quality of the materials. The findings of this study will provide standards for future commercialization, avoiding waste and sustainable environmental protection of the earth’s resources.

## 2. Materials and Methods

### 2.1. 3D Printing Machine and Printing Materials

In this study, the X1E 3D printer X1E produced by INFINITY 3DP Co., Ltd. (Kaohsiung, Taiwan) was used. The specifications of the 3D printer are shown in Table 1. The material used in this test was PLA supplied by Min-Yau Information Co., Ltd. (New Taipei, Taiwan). The material of wire diameter was 1.75 ± 0.05 mm. Before printing, the PLA material was placed in a humidity control box to keep them dry. The specifications of the printing materials are shown in Table 2.

### 2.2. Print Procedure and Parameters

**Figure 1:**
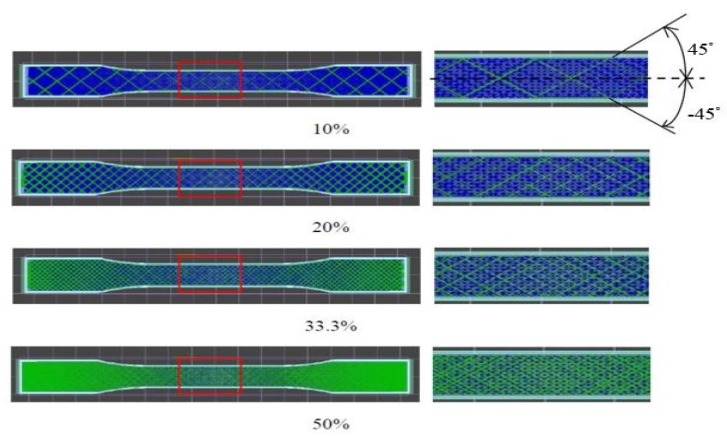
A schematic view of each filling percentage.

First, the Autodesk Inventor software (Autodesk Inc., San Rafael, CA, USA) was used to draw 3Dpictures of the test specimens. Since most slicing software accepts .stl files and .obj files, the Inventor files were converted to .stl format. Then the file was delivered to the slicing software for parameter adjusting. KiSSlicer was used as the slicing software. The experimental factors of the slicing software were categorized into control factors and fixed factors. The fixed factors included printing speed of 37.5 mm/s, platform temperature of 60 °C and the rectilinear type of filling pattern. The raster angle of 45°/45° was chosen, as shown in Figure 1. The controlling factors included filling percentages of 10%, 20%, 33.3%, and 50%, and printing nozzle temperatures of 185 °C, 195 °C, 205 °C, 215 °C, and 225 °C. To minimize the effect on the results of the experiment, this experiment was mainly based on the parameters set by the slicing software, as shown in Table 3. The quality of a 3D-printed object depends on the quality of the initial 3D model of the object; therefore, the image acquisition step was essential for the quality of a 3D-printed object. The three different requirements of printing quality were: (i) low surface roughness, (ii) good mechanical properties, and (iii) short printing time. This study discusses the mechanical properties of printing quality.

### 2.3. The Tensile Test

**Figure 2:**
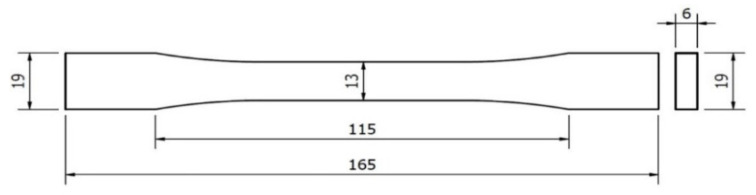
The ASTM D638 tensile test (size: mm).

The tensile test was performed in accordance with ASTM D638, as shown in Figure 2. The mechanical properties of the material were obtained and measured by a universal testing machine (model: YM-H5101) produced by Yang Yi Technology Co., Ltd. (Tainan, Taiwan). The specifications of the machine are shown in Table 4. The test speed was fixed at a rate of 5 mm/min, the maximum load was 100 kN with the signal sampling rate of 5 Hz. The elongation, force load, and ultimate tensile strength data were recorded by the built-in software. For each type of parameter of the specimens, five identical specimens were produced and tested. The average values of the quadruplicate of the individual parameters were selected. The obtained data from the experiment were analyzed by EXCEL software. This study mainly discusses the rate of change of mechanical properties. Therefore, the definition of the rate of change of Young’s modulus ($\dot{E_{ij}}$), ultimate tensile strength ($\dot{\sigma_{u}}$), and the tensile elongation (${\dot{\delta }}_{ij}$) are shown in the following Formulas (1)–(3):

(1) $$\dot{E_{ij}}=\frac{{\left(E_{ij}\right)}_{uv}-{\left(E_{ij}\right)}_{Green}}{{\left(E_{ij}\right)}_{Green}}\times 100\%,$$

(2) $$\dot{\sigma_{ij}}=\frac{{\left[{\left(\sigma_{u}\right)}_{ij}\right]}_{uv}-{\left[{\left(\sigma_{u}\right)}_{ij}\right]}_{Green}}{{\left[{\left(\sigma_{u}\right)}_{ij}\right]}_{Green}}\times 100\%,$$

(3) $${\dot{\delta }}_{ij}=\frac{{\left(\delta_{ij}\right)}_{uv}-{\left(\delta_{ij}\right)}_{Green}}{{\left(\delta_{ij}\right)}_{Green}}\times 100\%,$$

where, ${\left(E_{ij}\right)}_{uv},\;{\left[{\left(\sigma_{u}\right)}_{ij}\right]}_{uv},\;$ and ${\left(\delta_{ij}\right)}_{uv}$ are the tensile Young’s modulus, ultimate tensile strength and tensile elongation of the material after UV curing; ${\left(E_{ij}\right)}_{Green},\;{\left[{\left(\sigma_{u}\right)}_{ij}\right]}_{Green},\;$ and ${\left(\delta_{ij}\right)}_{Green}$. are the tensile Young’s modulus, ultimate tensile strength, and the tensile elongation of the raw material. *i* = 1, 2, 3, 4, 5, *j* = 1, 2, 3, 4; the meaning of each item number is shown in Table 5.

### 2.4. The Hardness Test

A model MET-DHG-D Shore D hardness tester produced by S.E.A.T Industry Technology Co., Ltd. (Kaohsiung, Taiwan) was used to analyze the hardness of specimens. The diameter of the indenter was 0.1 mm. The depth of the indenter was up to 2.5 mm. The specifications of the hardness tester are shown in Table 6. Shore D is an indentation hardness physical test with a steel indentation needle. Under the applied force, it was pressed vertically into the surface of the test specimen. When the surface of the indenter was fully attached to the surface of the material, the process was stopped for one second to read the data and record the test specimen. The average value of four times among five times measurements was taken. This experiment explored the rate of change of hardness. The formula for the rate of change of hardness (${\dot{H}}_{ij}$) is shown as (4). The measurement range was 10 mm up and down in the breaking direction of the tensile test specimen:

(4) $${\dot{H}}_{ij}=\frac{{\left(H_{ij}\right)}_{uv}-{\left(H_{ij}\right)}_{Green}}{{\left(H_{ij}\right)}_{Green}}\times 100\%,$$

where, ${\left(H_{ij}\right)}_{uv}$ and ${\left(H_{ij}\right)}_{Green}$ represent the hardness value of the material with and without UV curing process respectively. *i* = 1, 2, 3, 4, 5, *j* = 1, 2, 3, 4; the meaning of each item number is shown in Table 5.

### 2.5. The UV Curing Test

After the test specimens were fabricated by the 3D printer, the specimens were put into a MultiCure 180 curing machine produced by XYZ Printing, Inc. (Taipei, Taiwan) for UV curing for 60 min with a wavelength of 425 nm. The specifications of the curing machine are shown in Table 7. The fixed factors adopted included the energy intensity of 60%, the curing time of 60 min, and the rotation speed of the turntable of 1 circle per min.

## 3. Results and Discussion

**Figure 3:**
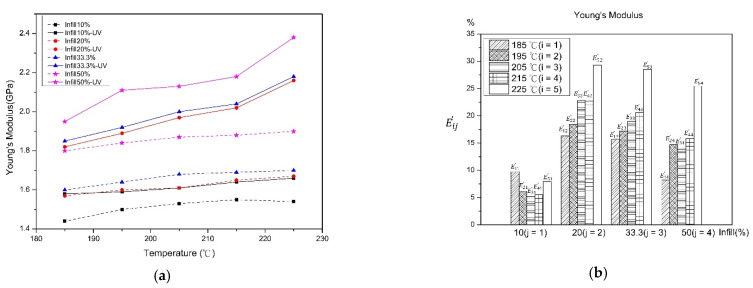
Before and after UV curing (**a**) Young’s modulus (**b**) rate of change.

The result values and the rate of change of tensile modulus are presented in Figure 3, according to which, the value of Young’s modulus increased with the increase in the infill percentage. This is because the air gaps of the cross-section of specimens are reduced with the increasing infill percentage. The enhancement of the contact area between layers promotes rigidity of the PLA material [8]. Another reason may be that the higher printing temperature allows the material to have enough fluidity to enhance the interfacial bond strength between layers, moreover, the interchain interaction and numbers of air gap also reduce, which increase the rigidity of material [10,25]. The rising trend of rigidity after the UV curing process is due to the improved combination of crosslink by UV irradiation. The reason for reduction of change of rate of Young’s modulus with the decreasing infill percentage is because the lower infill percentage causes the sparse inner structure of specimens, which makes the penetration of the UV light to the layers and deforms the material. Furthermore, increasing the printing temperature also increases the rate of change of Young’s modulus, because the higher temperature enhances the combination of intermolecular melting material, which provides a better ability of the UV light to conduct the energy from the interface of material to inner structure, therefore, resulting in a better combination between layers result in the higher rigidity of the material.

**Figure 4:**
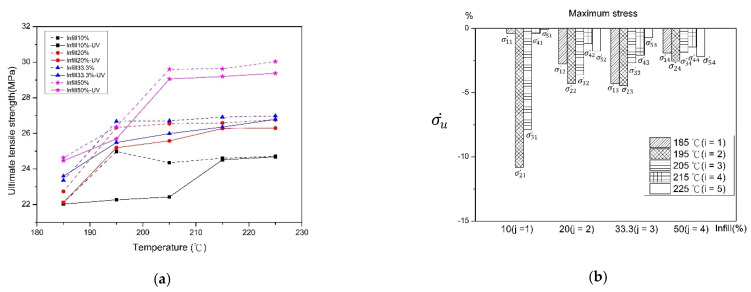
Before and after UV curing (**a**) The ultimate tensile strength (UTS), and (**b**) the rate of change.

The value and the rate of change of ultimate tensile strength (UTS) with and without the UV curing process are shown in Figure 4. An uptrend of UTS was observed with the increasing infill percentage and printing temperature. This is because the higher infill percentage causes tightening of the inner structure, which enhances the toughness of the material [26]. Higher printing temperatures provide enough fluidity and reduce the adhesion of melting PLA, which increases the dynamic speed of material and causes energy transfer into the deposited layer. When the adhesion between layers increases, the intensity of PLA is also enhanced [21,25]. A slight downtrend of tensile strength is also seen after the UV curing process. This is because the UV light irradiation embrittles the material, which renders the specimens prone to failure [27]. As the filling percentage increases, the change in the rate of intensity decreases. Due to the low filling percentage, the internal density of the test specimen is lower, and UV light can easily penetrate to the next layer to transmit energy to a deeper layer. As a result, the poor layer-to-layer bonding and the brittleness properties of the material lead to easy breakage. The rate of change in tensile strength increases with the decrease in printing temperature because the lower printing temperature causes the incomplete melting of the material, which reduces the layer-to-layer adhesion and exhibits brittleness.

**Figure 5:**
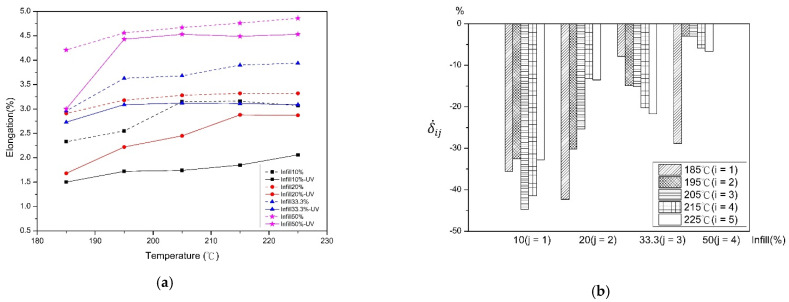
Before and after UV curing (**a**) elongation and (**b**) the rate of change.

Figure 5 shows the elongation value and rate of change before and after UV curing. It can be observed that as the filling percentage increases, the elongation also increases. Since FDM is a discontinuous process, the discontinuity properties decrease as the filling percentage increases, which reduces the void structure between layers. The enhancement of the contact area increases the elongation of PLA materials [13]. As the printing temperature increases, the elongation also increases. The larger material fluidity caused by high-temperature printing leads to the reduction of the interaction between material chains and the reduction of interlayer voids, thus improving the toughness of PLA materials [21]. After the UV curing process, the ductility of the material is significantly reduced. The reason may be because, after UV irradiation, the material chains are closer together, leading to rapid aging and brittleness. As the filling percentage increases, the elongation change rate decreases. It is shown that the smaller the filling percentage, the sparser of the density between layers and the larger the numbers of air gaps in the specimens. The PLA material irradiated by UV light with a larger wavelength for a short time results in rapid aging of the material, with brittle properties of the material. It can be seen that the lower printing temperature reduces the change in the rate of elongation. This is because the low-temperature printing causes poor bonding between layers. After UV curing process, the bonding between layers is slightly strengthened, resulting in a large change in the material.

**Figure 6:**
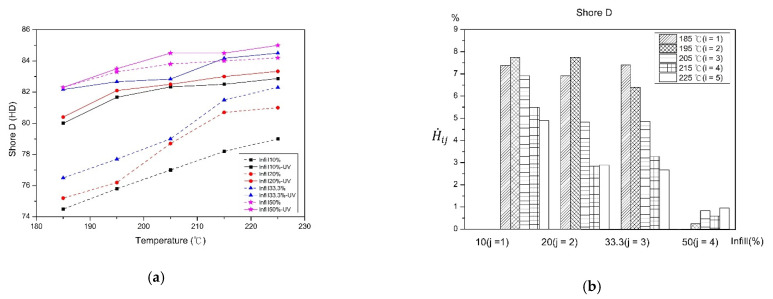
Before and after UV curing (**a**) hardness and (**b**) the rate of change.

Figure 6 shows the hardness value and rate of change before and after UV curing. As the filling percentage increases, the value of hardness also increases. As the percentage of filling increases, the cross-sectional area of the material increases, resulting in a harder internal structure. With the decrease in the printing temperature, the hardness value also decreases. When printing at low temperature, the material melts incompletely, the layer-to-layer bonding is poor, so it produces larger porosity and lower hardness. A significant increase in the hardness of the material is observed after UV curing. After the material is UV irradiated, it leads to a closer distance of material chains. As the filling percentage increases, the rate of change of hardness decreases. The larger air gap of the internal structure appears on the low filling percentage, which makes the pressure needle to easily penetrate the surface of the test specimen. After the test specimen is irradiated by the UV light, the molecular chains on the surface of the material are rearranged more tightly, which increases the hardness of the test specimen after curing. As the printing temperature increases, the rate of change in hardness decreases, this may because the high printing temperature or the work of adhesion between fibers causes a stronger combination between the material [28], and the numbers of air gaps are also reduced. Another reason may that the raw material has high hardness after penetrating to the next layer by UV light, which slightly enhances the bonding between layers, and leads to a slight increase in the rate of hardness.

**Figure 7:**
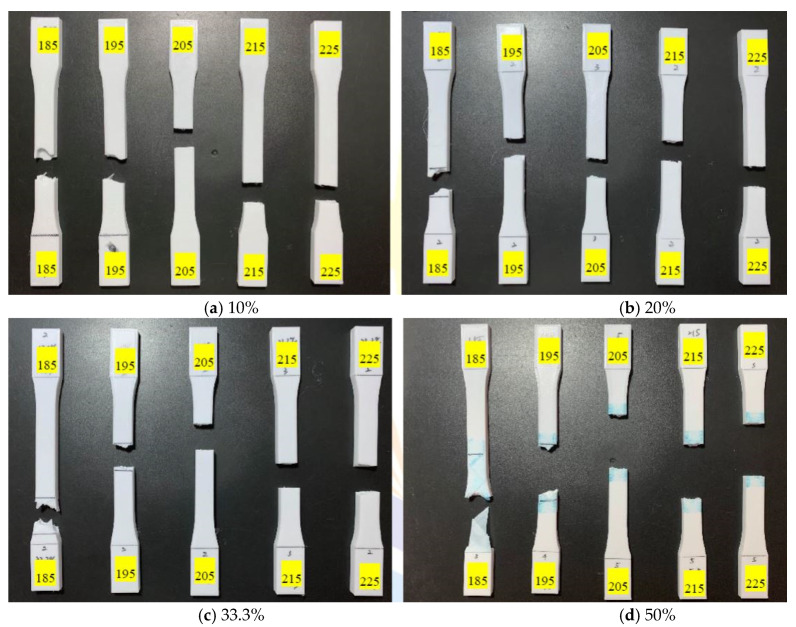
Tensile test specimens with different filling percentages (**a**) 10%, (**b**) 20%, (**c**) 33.3%, and (**d**) 50% under various printing temperature conditions before UV curing.

**Figure 8:**
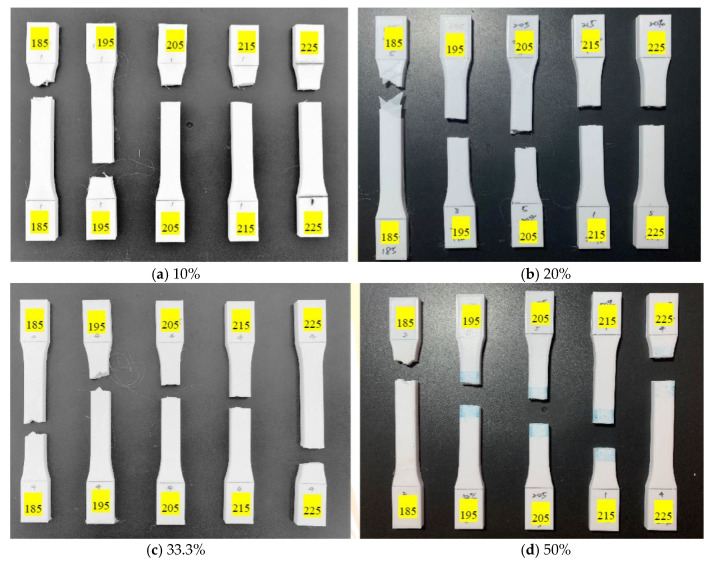
Tensile test specimens with different filling percentages (**a**) 10%, (**b**) 20%, (**c**) 33.3%, and (**d**) 50% under various printing temperature conditions after UV curing.

Figure 7 and Figure 8 demonstrate the image of tensile test specimens with and without UV curing, respectively. Table 8 shows the CCD image of surface morphology of the tensile test specimen before and after UV curing. Figure 7 and Table 8 show that as the printing temperature increases, the fibers and the appearance of specimens become tightened and flattened. The breaking direction of the test specimen at the printing temperature of 185 °C and 195 °C is along with the printing pattern (Figure 1). When the printing temperature is more than 195 °C, the fracture direction of the test specimen is vertical to the tensile applied direction. An incomplete melting due to the lower printing temperature causes poor bonding between layers and the loss of internal structure [29]. The two failure modes are defined as inter-failure mode and in-layer failure mode [30]. The inter-layer failure mode causes the test specimen to break from the area in the printing direction of the material [8]. The high-temperature printing material has a better internal bond, which causes an in-layer failure mode. Figure 8 and Table 8 show an aging phenomenon of the PLA after UV light irradiation, resulting in a relatively uneven state between the surface fibers. As the temperature of the nozzle increases, the pores between the fibers are reduced, and a flatter appearance can be seen on the test specimen. At a lower printing temperature (185 °C), the surface fibers of specimens are tightened. However, the internal structure is loose, leading the specimens to fracture easily between layers. Figure 7 and Figure 8, and Table 8 show that the change of the filling percentage has no significant effect on the fineness of the surface topography and the direction of fracture.

## 4. Conclusions

This study explored the effect of printing parameters, including nozzle temperature and infill percentage, on mechanical properties of materials produced based on FDM. The tensile and hardness properties of PLA specimens with and without the UV curing process were investigated. The findings of this research suggest that changing the printing parameters has the following effect on mechanical properties:

(1) Increasing ease in the infill percentage and the nozzle temperature enhance the Young’s modulus and Shore hardness of materials about 23% and 2.3%, respectively.
(2) The strength and the elongation of the material increase about 16% and 61%, respectively, with the nozzle temperature and the infill percentage but decrease after the UV curing process.
(3) After the UV curing process, there is a significant reduction in the rate of change of strength, and elongation about 2.2% and 6.79%, respectively, but the rate of change of Young’s modulus increases about 25.26%.
(4) The materials printed at low temperature have in-layer failure mode, while those printed with high temperature have inter-layer failure mode. The same phenomenon was also seen in the material after the UV curing process.
(5) Increasing the nozzle temperature results in a smooth and close exterior of specimens. The infill percentage has no significant effect on the exterior of specimens with and without UV curing process.

## Figures and Tables

**Table 1:** The specifications of the 3D printer.

Name	Specification
Physical dimension	(w) 40 cm × (d) 22 cm × (h) 46 cm
Maximum printing area	(w) 21 cm × (d) 21 cm × (h) 24 cm
Print layer height	0.04~0.32 mm
Nozzle diameter	0.2–0.8 mm
Wire diameter	Φ1.75 mm
Platform temperature	0~110 °C
Nozzle printing temperature	0~280 °C
Cooling method	4.5 cm turbofan
Printing speed	0–100 mm/s
Motor drive	1/32 micro-stepping motor (8825 driver chip)
X/Y motion mechanism	Guideway

**Table 2:** The specifications of the PLA.

Name	Specification
Material	PLA
Color	Snow white
Wire diameter	1.75 ± 0.05 mm
Fan	Open
Weight	800 g
Platform temperature	60 °C
Recommended printing speed	30~50 mm/s
Recommended printing temp	185~215 °C

**Table 3:** Process parameter conditions of the printing material.

	Content	Name	Range
Project			
Fixed factor		Material	PLA (snow white)
		Printing speed	37.5 mm/s
		Layer thickness	0.2 mm
		Platform temperature	60 °C
		Nozzle diameter	0.4 mm
		Raster angle	45°/−45°
		Printing pattern	rectilinear
		Retraction	120 mm/s
Controlling factor		Printing temperatures	185 °C, 195 °C, 205 °C, 215 °C, 225 °C
		Infill densitiesdensities	10%, 20%, 33.3%, 50%

**Table 4:** The specifications of the YM-H5101 universal testing machine.

Name	Specification
Model	YM-H5101
Size	(w) 120 cm × (d) 65 cm × (h) 220 cm
Weight	800 kgf
Capacity	100 kN
Count	0–100
Displacement resolution	0.001 mm
Stroke	1100 mm (without fixtures)
Motor	Servo motor
Sampling rate	5–500 Hz
Load resolution	1/10,000–1/200,000
Speed	0.003–375 mm/min

**Table 5:** The definition of number *i* and *j* of the change rate of the printing temperature and infill density respectively.

Item	Parameters	1	2	3	4	5
*i*	Printing temperature (°C)	185	195	205	215	225
*j*	Infill density (%)	10	20	33.3	50	

**Table 6:** The specifications of the Shore D.

Name	Specification
Model	Shore D
Size	(L) 88 mm × (w) 57 mm × (d) 25 mm
Indenter diameter	0.1 mm
Indenter depth	0–2.5 mm
Weight	199 g
Range	0~100 HD
Count	At least three times
Shape	Cone
Material	Steel

**Table 7:** The specifications of the MultiCure180.

Name	Specification
Model	MultiCure180
Size	(w) 255 mm × (d) 255 mm × (h)352 mm
UV wavelength	UV LED λ 355 nm, 405 nm, 425 nm
Range	∅180 mm × (h) 200 mm
Temperature	0~60 °C
Time	1–60 min
Turntable speed	1 circle/1 min (fixed)
Weight	1.5 kg
Energy intensity	0~100%

**Table 8:** Surface morphology of the specimens before and after UV curing.

	**185 °C**	**195 °C**	**205 °C**	**215 °C**	**225 °C**
10%	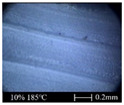	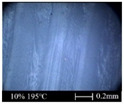	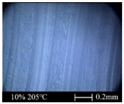	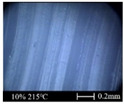	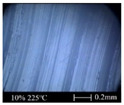
UV-10%	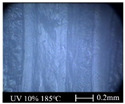	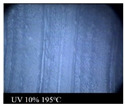	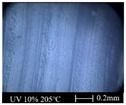	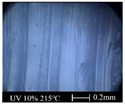	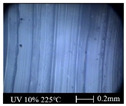
20%	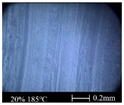	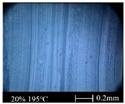	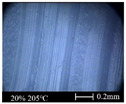	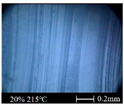	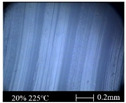
UV-20%	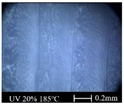	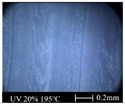	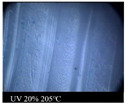	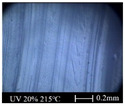	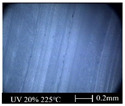
33.3%	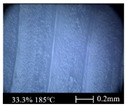	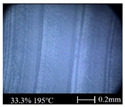	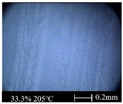	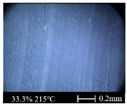	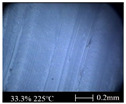
UV-33.3%	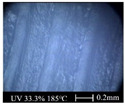	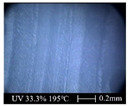	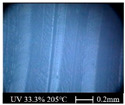	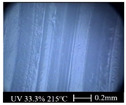	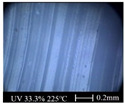
50%	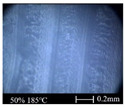	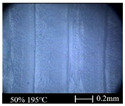	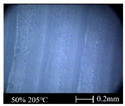	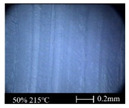	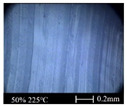
UV-50%	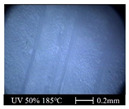	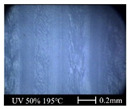	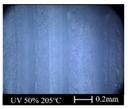	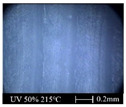	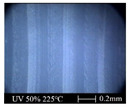

## Data Availability

Not applicable.
